# Strategies in the development of pro-oxidant therapy for oral squamous cell carcinoma: A scoping review

**DOI:** 10.1016/j.jtumed.2025.06.002

**Published:** 2025-06-27

**Authors:** Zainab Niazi, Hassan Mujtaba, Nurul R. Ibrahim, Mogana D. Murtey, Norhayati Yusop

**Affiliations:** a Basic Sciences Unit, School of Dental Sciences, Universiti Sains Malaysia, Malaysia; b Department of Oral Pathology, Islamabad Medical and Dental College, Pakistan; c Department of Oral Pathology, School of Dentistry, Shaheed Zulfiqar Ali Bhutto Medical University, Islamabad, Pakistan; d Department of Maxillofacial Surgery and Oral Diagnosis, Kuliyyah of Dentistry, International Islamic University of Malaysia, Malaysia

**Keywords:** سرطان الخلايا الحرشفية الفموي, مادة مؤكسدة, علاج, إدارة, معالجة, Management, Oral squamous cell carcinoma, Pro-oxidant, Therapy, Treatment

## Abstract

**Background:**

Pro-oxidant therapy has gained attention as newly potential approach in combating oral cancer. However, there are lack of classification and comparative analysis on extensive literatures which focuses on the different strategies and efficacy of pro-oxidant based therapies. This review aims to investigate the development of pro-oxidant therapy in treatment of oral cancer, emphasising on the fundamental knowledge behind each method, the impacts of each pro-oxidant-based method on cancer cell lines, and the future application in clinical practices.

**Method:**

The present scoping review is carried out according to the Arksey and O'Malley framework's four-stage scoping review technique. The search was conducted from different electronic research databases such as Google Scholar, Web of Science, PubMed, and Scopus to select relevant peer-reviewed studies. The search strategy includes keywords such as “strategies” “development”, “pro-oxidant”, therapy”, “oral”and “squamous cell carcinoma”. The articles were screened, by keeping in mind the inclusion and exclusion criteria, along with assessment for eligibility. The present study is registered with Open Science Framework (OSF), followed by PRISMA-ScR guidelines to increase the relevance for decision-making.

**Results:**

313 articles were selected by searching the literature databases, 250 were identified relevant to the present scoping review topic. Following the screening criteria, only 15 articles were included in the final review for detail analysis.

**Conclusions:**

The gathered data positively contribute to more discoveries of advanced techniques for the management of cancer with minimal toxicities to improve on treatment outcomes.

## الملخص

**أهداف البحث:**

اكتسب العلاج ماقبل الأكسدة اهتماما مؤخرا كنهج محتمل في مكافحة سرطان الفم. تتيح مراجعة نطاق البحث تصنيفا وتحليلا مقارنا لأدبيات واسعة تركز على الاستراتيجيات المختلفة المستخدمة في العلاجات ماقبل الأكسدة. تهدف الدراسة الحالية إلى دراسة الاستراتيجيات المختلفة المستخدمة لتطوير العلاج ماقبل الأكسدة في علاج سرطان الفم، وتحديدا سرطان الخلايا الحرشفية الفموي، مع التركيز على المعرفة الأساسية وراء كل نهج، وتأثيرات كل طريقة قائمة على ماقبل الأكسدة على سلالات الخلايا السرطانية، والتطبيق المستقبلي في الممارسات السريرية.

**طريقة البحث:**

أجريت مراجعة نطاق البحث الحالية وفقا لتقنية مراجعة نطاق البحث المكونة من أربع مراحل لإطار عمل أركسي وأومالي. أجري البحث من قواعد بيانات بحثية إلكترونية مختلفة مثل جوجل سكولار، وويب أوف ساينس، وبوب ميد، وسكوبس لاختيار الدراسات ذات الصلة التي خضعت لمراجعة الأقران. تتضمن استراتيجية البحث مجموعة من الكلمات المفتاحية المتعلقة بالموضوع باستخدام مصطلحات مثل "الاستراتيجيات"، و"التطوير"، و"مادة مؤكسدة"، و"العلاج"، و"الفموي"، و"سرطان الخلايا الحرشفية". تم فرز المقالات مع مراعاة معايير الإدراج والاستبعاد، بالإضافة إلى تقييم الأهلية. الدراسة الحالية مسجلة في إطار العلوم المفتوحة، وتخضع لإرشادات بريزما-اس سي آر لزيادة ملاءمتها لاتخاذ القرارات.

**النتائج:**

تم اختيار ٣۱٣ مقالة من خلال البحث في قواعد بيانات الأدبيات، وتم تحديد ۲٥۰ مقالة ذات صلة بموضوع مراجعتنا النطاقية. باتباع معايير الفرز، تم تضمين ۱٥ مقالة فقط في المراجعة النهائية لتحليلها بالتفصيل.

**الاستنتاجات:**

قد تساهم البيانات المجمعة من هذه المراجعة بشكل إيجابي في اكتشاف المزيد من التقنيات المتقدمة لإدارة السرطان، مع الحد الأدنى من السمية لتحسين نتائج العلاج.

## Introduction

Oral squamous cell carcinoma (OSCC) is the most common and aggressive type of cancer that arises from the squamous epithelium in the oral cavity. It has a high morbidity and mortality rate, and more than 90 % of oral cancers are diagnosed as OSCC.^1^ A poor prognosis has been reported for OSCC due to aggressive tumor behavior, late stage diagnosis, and frequent recurrences despite advances in conventional treatment methods such as radiation, surgery, and chemotherapy.^2^ Furthermore, the traditional therapeutic approaches, such as surgical procedures, radiation therapy, and chemotherapy, are frequently associated with major problems, including tumor relapse, metastasis, resistance, and immediate side effects.^3^ Pro-oxidant therapy aims to increase the levels of reactive oxygen species (ROS) in cancer cells to above a specific threshold in order to induce cytotoxicity and cell death.^4^ Due to their rapid metabolism and genetic instability, cancer cells typically exhibit higher ROS levels at baseline. Pro-oxidant therapy preferentially targets cancer cells by boosting ROS while sparing healthy cells. According to the existing evidence, new therapeutic approaches are desperately needed to increase the rate of survival and success for oral cancer patients.^5^ Pro-oxidant therapy based on the unique oxidative vulnerabilities of cancer cells is an effective strategy for this purpose.

At present, surgery is the primary approach for treating OSCC, particularly in cases of locally advanced cancers. Surgical procedures vary from simple excision to complicated methods that require dissection of the jawbone and neck to remove lymph nodes.^6^ Advances in surgical techniques, such as the use of reconstructive microvascular free flaps, have significantly improved the functional and aesthetic outcomes for patients. However, in late stage cases of OSCC, surgery is typically insufficient and additional therapeutic techniques are required.^7^ After surgery, radiation therapy is commonly used as an adjuvant treatment to eliminate any residual cancer cells.^8^ Modern techniques such as intensity-modulated radiation therapy can reduce the damage caused by radiation to surrounding healthy tissues by increasing the accuracy of radiation administration.^9^ Radiation therapy also has serious side effects, including mucositis, osteoradionecrosis, and xerostomia.^10^

In addition, chemotherapy is frequently used to treat advanced or metastatic OSCC to reduce tumors and slow the disease’s progression by using common chemotherapy drugs, such as fluorouracil, cisplatin, and taxanes.^11^ However, the development of drug resistance and serious side effects restrict the long-term effectiveness of chemotherapy. Immunotherapies and targeted treatments have obtained better results in the treatment of OSCC in recent years.^12^ Immune checkpoint inhibitors that target specific molecular pathways, such as pembrolizumab and epidermal growth factor receptor (EGFR), have numerous advantages in terms of clinical practice.^13^ These therapies inhibit important pathways for tumor survival and growth as well as improving the immune system’s defenses against cancer cells.^14^ However, the wide variety of malignancies and development of resistance, may restrict the efficacy of these therapeutic agents.^15^

The progression and initiation of OSCC are often associated with the accumulation of ROS, which are produced by redox reactions.^16^ A high metabolic rate, hypoxia, and gene mutations are known to produce high levels of ROS in tumor microenvironments.^17^ ROS includes an extensive range of radical and non-radical reactive species that affect major signaling pathways, including MAPK/ERK1/2, nuclear factor-kappa B (NF-κB), and PIK3/Akt.^1^ Dysregulated redox homeostasis in the tumor microenvironment affects the physiology of cell division, and is closely associated with the progression of cancer. Elevated levels of ROS can damage lipids, proteins, and DNA, and cause cell death. Cytochrome C may be released from mitochondria because of the presence of ROS to activate caspases and trigger apoptosis. Elevated ROS levels can lead to protein misfolding and stress in the endoplasmic reticulum, leading to apoptosis.^18^

Pro-oxidant therapy has attracted attention as a potential cancer treatment based on exploiting the elevated levels of oxidative stress in cancer cells.^15^^,^^16^ In practice, pro-oxidant therapy efficiently targets cancer cells while safeguarding healthy cells by elevating ROS levels. In the present review, we provide an overview of the different strategies used in the development of pro-oxidant therapy for OSCC over the years, particularly the effects of this therapy on cancer cell lines, the mechanisms involved, and possible therapeutic implications. This information may be used for efficiently optimizing the levels of ROS in cancer cells to exceed the thresholds that cause cell death and cytotoxicity, with a higher likelihood of positive outcomes.

## Materials and Methods

This scoping review was performed by identifying relevant publications. We adhered to Arksey and O’Malley’s four-stage scoping review framework for this review. The five stages in this review process are as follows.

## Stage 1: identification of research questions

The PCC framework was selected to focus on the research questions. PCC denotes “Population, Concepts, and Context” in the PCC framework. For this review, “Population” refers to individuals suffering from OSCC, “Concepts” are pro-oxidant treatments, strategies for treatment, and techniques for management and settings, and “Contexts” refers to the current practices, and efficiency and standardization of implementation.

## Research questions

The present review was performed to address the following questions:

a) What are the current strategies used in the development of pro-oxidant therapies for OSCC treatment and management?
b) How successful are these strategies at enhancing patient outcomes?

These questions were formulated in order to explore the various approaches used in pro-oxidant therapy for OSCC, to assess their effectiveness, and to examine how these strategies can be standardized to enhance patient care.

## Stage 2: identifying relevant studies

Four electronic research databases were searched to select relevant peer-reviewed studies for this scoping review: Google Scholar, PubMed, Web of Sciences, and Scopus. The search strategy was based on the following keywords related to the topic: “strategies,” “development,” “pro-oxidant,” “therapy,” and “oral squamous cell carcinoma.” Table 1 shows the specific search strings used in the four databases.

**Table 1:** Search strings used to identify relevant studies in four databases.

Database	Search String
**PubMed**	(“Pro-oxidant therapy” OR “oxidative stress”) AND (“OSCC” OR “oral squamous cell carcinoma”)
**Scopus**	(“Pro-oxidant agents” AND “cancer therapy” AND “reactive oxygen species”)
**Web of Science**	(“Nanotechnology-based therapy” OR “stem cell-based therapy” OR “Phytocompounds-based therapy” OR “radiation therapy”) AND (“oral cancer”)
**Google Scholar**	“Pro-oxidant therapy in OSCC treatment”

## Stage 3: study selection

The articles were screened according to inclusion and exclusion criteria, and assessed to determine their eligibility according to the accessibility of full-text articles. Free and accessible full-text articles were downloaded in PDF format, and restricted articles were obtained via the institutional access of Universiti Sains Malaysia. The inclusion criteria included articles where the primary objective was pro-oxidant therapy interventions for the treatment or management of OSCC, and the exclusion criteria were as follows:

i. Studies describing cancers other than OSCC.
ii. Articles that focused on treatments other than pro-oxidant therapy as a primary treatment modality for OSCC.
iii. Editorials, non-research articles, and opinion pieces.
iv. Abstracts without full-text articles.
v. Articles in languages other than English.
vi. Studies published longer than 12 years ago.
vii. Studies that lacked sufficient data regarding the implementation of pro-oxidant therapy or the outcomes for treatment and management of OSCC.

## Stage 4: extraction of publications

Two independent authors completed the final data extraction process for the papers included in a comparative manner. The authors' names, title, publishing date, and key information relevant to the topics and treatment options were extracted. Each identified record was further examined through backward citations searching (snowballing) to search for gray literature sources. A thorough examination was performed of the reference lists for a known set of relevant articles as the starting point to uncover articles that might have been missed in the initial database search. The reference list reviewing process was performed manually. Findings from gray literature, which refers to poorly indexed databases such as reports, conference proceedings, theses, and other non-commercial publications, were appropriately documented, particularly if the studies provided valuable insights and perspectives not found in peer-reviewed journals, although gray literature was excluded at the end of the reviewing process.

## Stage 5: synthesis of results

The authors' names, year, interventions, outcomes, and significant findings gathered from citations were all compiled in a tabular data format. De-duplication of selected references was performed using Endnote™.

## Work outline

**Figure 1:**
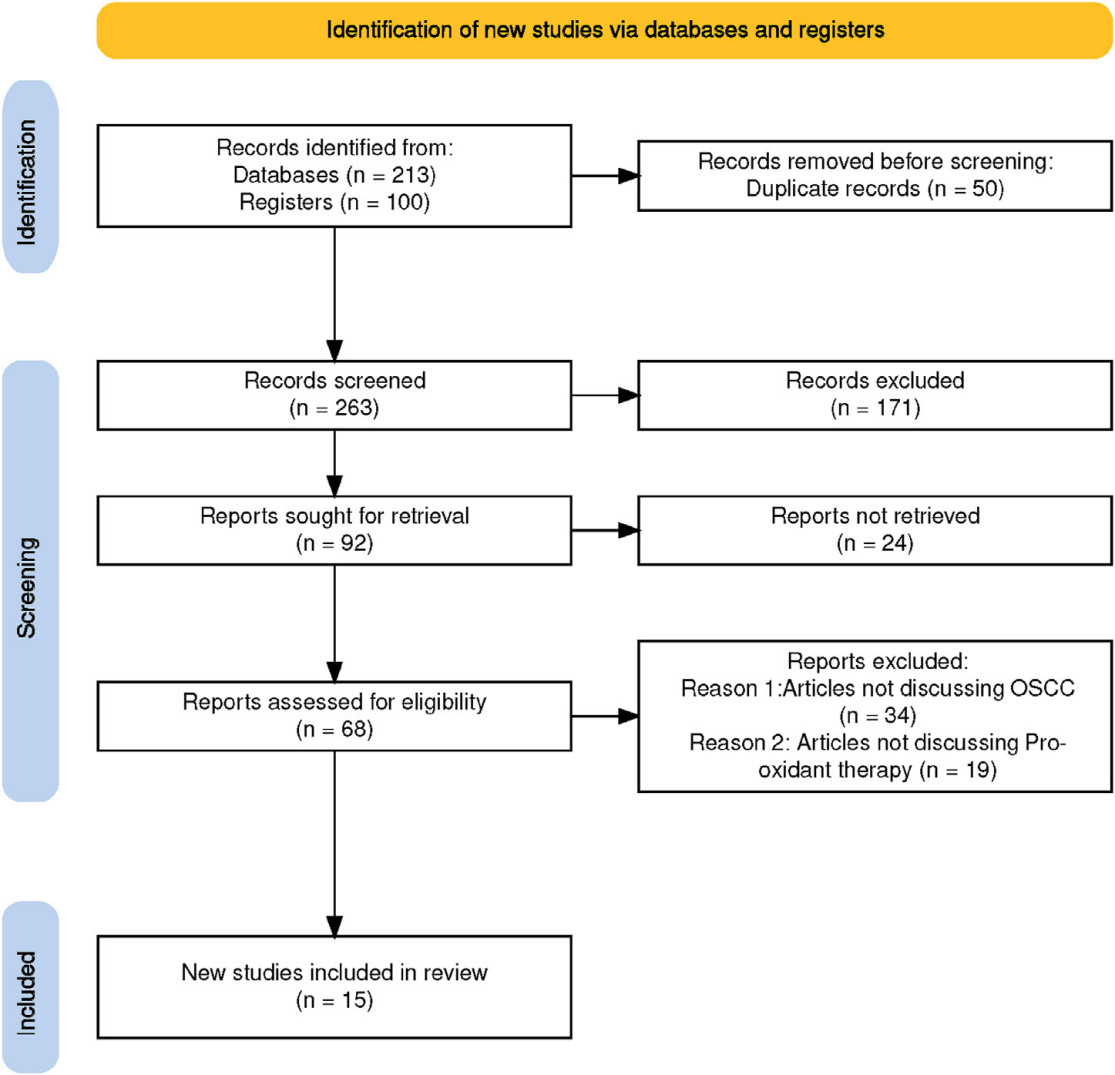
PRISMA-ScR flow chart.

The present review has been registered with the Open Science Framework (OSF). OSF is an open-access online database for sharing scientific protocols on a wide range of topics in order to prevent duplication or overlap with other researchers who might address the same problem statement and objectives. The PRISMA-ScR guidelines (Figure 1) were used to improve the completeness of reporting, facilitate evaluation of the results, and to ensure that the information obtained was more relevant for making decisions. In addition, the following risk of bias (RoB) tools were used to assess the quality of different study types for this scoping review: Cochrane’s Risk of Bias 2.0 (RoB 2) for clinical randomized controlled trials (RCTs); ROBINS-I tool for non-RCTs; SYRCLE’s RoB tool for preclinical animal studies; and the ToxR tool for *in vitro* studies. These tools helped to identify potential sources of bias that could have affected the reliability of our findings.

## Results

Our comprehensive literature search performed according to the stated criteria identified a total of 313 articles. Two separate reviewers conducted the screening process and 263 articles remained for further analysis after removing duplicates using Endnote™. Any record that satisfied the eligibility requirements was subsequently included in the study. The articles were then screened based on their titles and abstracts, resulting in 68 articles for detailed evaluation based on the specific criteria. After reviewing the full texts, 53 articles were excluded based on the established eligibility criteria. Any differences of opinion between the reviewers were resolved by discussion with a third reviewer. After scanning the gray literature database and conducting the citation search, 15 articles were finally selected.

## Categories of pro-oxidant therapies for OSCC

**Figure 2:**
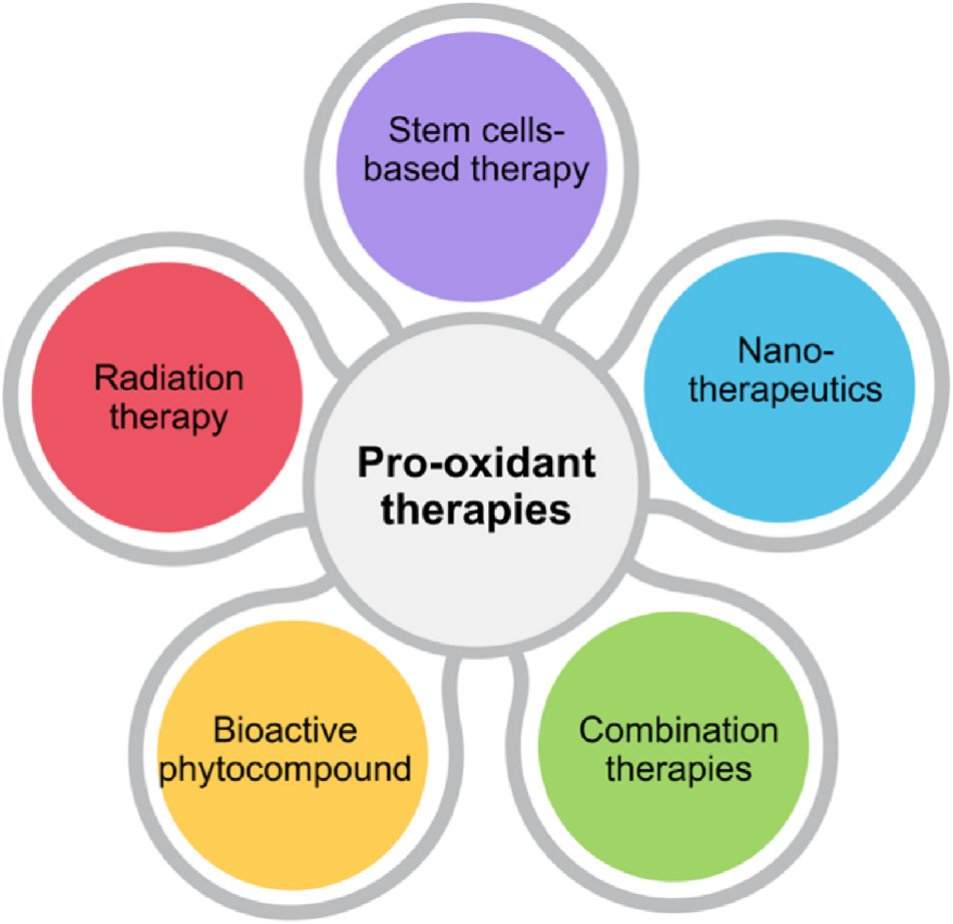
Current types of pro-oxidant therapies used for OSCC.

According to the 15 selected articles, five different types of pro-oxidant therapies are targeted for the development of therapy and management of OSCC. These five types of strategies are nanotechnology-based therapy, stem cell-based therapy, phytocompound-based therapy, radiation therapy, and combination therapy, as illustrated in Figure 2.

## Types of pro-oxidant agents for OSCC

ROS are by-products of metabolic reactions that enhance various cellular processes. However, high levels of ROS result in oxidative stress, which can then cause cell death. It has been established that the redox status plays a significant role in determining the fate of cancer cells. Compared with normal cells, cancer cells are more susceptible to damage caused by oxidative stress. Therefore, pro-oxidants have attracted significant interest as potential chemotherapeutic agents in recent years. To increase intracellular ROS concentrations, pro-oxidant therapies can be applied by either directly producing ROS or by specifically targeting and blocking the natural antioxidant systems in cancer cells. This concept has led to the development of pro-oxidant-based therapy. The ultimate aim is to increase ROS levels in cancer cells to induce oxidative stress and cell death. Table 2 shows the pro-oxidant agents studied specifically for the treatment of OSCC according to the selected records.

**Table 2:** Pro-oxidant agents tested in laboratory experiments and pre-clinical studies to develop therapies for OSCC.

Material tested	Method used	Mechanism	Reference
Curcumin	*In vitro*	Restore status of lipid peroxidation, antioxidants, and ROS	19
Ferulic acid	*In vitro*	Modulate lipid peroxidation, carcinogen-detoxifying agents, and antioxidants	20
Quercetin	*In vitro*	↓ cell growth and invasion/migration of SCC-25 cells *in vitro*	21
Resveratrol	*In vitro*	↑ cell cycle arrest, ↑ apoptosis	22
Plumbagin	*In vitro*	↑ROS, ↓Bcl-2, ↑Bax, ↑CC3, ↑Beclin-1, ↓p62, ↑LC-II/LC-I, ↓p-AKT, ↓p-mTOR, ↑p-JNK	23
		↓ tumor cell proliferation, ↑ROS production, ER stress, mitochondrial dysfunction, and activation of caspases	24
Piperine	*In vitro*	↑ apoptosis due to ↓mitochondrial membrane potential and ↑ROS following cell cycle arrest and caspase-3 activation	25
Allicin	*In vitro*	Allicin induces apoptosis by ↑cascades of caspases as well as ↑p53 and Bax/Bcl2 expression	26
Arsenic trioxide	*In vivo/in vitro*	↑ proliferation, ↑apoptosis, ↓ angiogenesis, ↓ estrogen receptor signaling, and modulation of immune response	27
Melatonin	*In vitro*	↑ cisplatin, ↑GSSG/GSH, ↑Bax/Bcl-2, ↑NIX, ↑ATG12-ATG5	28
Metformin	*In vitro*	↑ ROS, ↓ME2, ↑p21, ↑NADP/NADPH, ↑SA-β-gal, ↑ radiation effect	29
d-allose	*In vitro*	↑ROS, ↑TXNIP, ↓TRX	30

## Bioactive phytocompounds for OSCC prevention and treatment

Natural agents including plant secondary metabolites or phytochemicals are very important in the prevention and treatment of cancer. Phytochemicals such as phenolics, terpenoids, alkaloids, and sulfur-containing compounds have anticancer actions against OSCC cells by regulating pathways including cytokine receptors and epidermal growth factor.^31^ *Curcuma longa* contains curcumin, which is a hydrophobic phenol that has anticancer effects by sensitizing OSCC cells to radiation by downregulating pro-survival proteins such as NF-κB and TxnRd1.^40^ Curcumin and its analogs exhibit encouraging anticancer activities against OSCC by increasing the formation of ROS, thereby leading OSCC cells to undergo apoptosis and autophagy.^19^ Intrinsic apoptosis occurs following the activation of caspases and disruption of the mitochondrial membrane potential by curcuminoids'.^41^ Curcumin also inhibits the expression of cyclooxygenase-2 and NF-κB in experimental induced OSCC.^19^ These findings suggest that curcumin and its derivatives could be good potential therapeutic agents for OSCC treatment.

Higher malic enzyme 2 (ME2) levels are linked to poor patient outcomes. However, ME2 expression is downregulated by metformin and ionizing radiation whereas ROS and p21 levels are elevated in OSCC.^48^ Melatonin (N-acetyl-5-methoxytryptamine), an FDA-approved chemical for dietary supplements, has anti-inflammatory, antioxidant, and anti-cancer effects.^32^ Hence, targeting ME2 could potentially improve therapeutic outcomes in OSCC cells and melatonin has been proposed as an adjuvant therapy to enhance *head and neck squamous cell carcinoma* outcomes, especially in cases where patients are resistant to standard treatments.^28^

Ferulic acid (FA) is a flavonoid compound with significant anticancer activities in various types of squamous cell carcinoma. In esophageal squamous cell carcinoma, FA increases ferroptosis by enhancing the production of ROS and the iron load, leading to reduced cell invasion and viability.^33^ In hamster buccal pouch carcinogenesis, FA exhibits chemo-preventive properties by modulating carcinogen-detoxifying agents and antioxidants, resulting in reduced tumor incidence and volume.^20^ Quercetin, another flavonoid compound, has anticancer activity against OSCC, where it decreases OSCC cell invasion, migration, and cell growth through different mechanisms that stimulate cell cycle arrest, especially in the G1 and S phases.^34^ Quercetin also causes apoptosis through caspase-3 activation and mitochondrial pathways. *In vivo* studies using hamster models have shown that quercetin decreases OSCC incidence by suppressing NF-κB signaling, and by modulating *Bcl-2* and *Bax* gene expression.^21^

Resveratrol, a natural polyphenol, has anti-proliferative and antioxidant properties in the treatment of OSCC, particularly in combination with chemotherapy. Injectable drug delivery devices loaded with resveratrol may be able to provide more focused care and obtain better results for clinical applications.^35^ In addition, *Plumbago zeylanica* contains plumbagin, which produces ROS, promotes apoptosis, and blocks important pathways such as NF-κB and PI3K/AKT/mTOR.^36^ Plumbagin decreases OSCC cell growth by enhancing ROS production, leading to mitochondrial dysfunction and caspase activation.^24^ Plumbagin increases the efficacy of cisplatin in tongue squamous cell carcinoma, and also increases intracellular ROS and inhibits AKT/mTOR signaling pathways.^23^

Piperine is another pro-oxidant agent that has attracted significant attention in recent years. Piperine is an alkaloid found in black pepper with strong anticancer properties against OSCC. Piperine causes OSCC cells to undergo apoptosis by activating caspase-3, lowering the mitochondrial membrane potential, and increasing the formation of ROS.^37^ Piperine was shown to slow the progression of buccal pouch carcinoma in hamsters through increasing antioxidant levels and detoxification enzyme activities.^38^ Another pro-oxidant agent that has been tested and widely used in clinical medicine is the small molecular compound d-allose. The use of d-allose is advantageous compared with other materials because it has proven therapeutic efficiency, few side effects, and a rapid absorption rate due to its small size, making it suitable for OSCC treatment.^39^

## Stem cell targeted therapy for OSCC

The rejuvenating effects of stem cells on aging and cancer development remain of particular interest in regenerative therapy. Stem cells, also known as pluripotent cells, are found in most self-renewing tissues, where they are characterized by the ability to maintain an undifferentiated state and their long-term auto-renewal capacity. Oxidative damage can affect all cells in an organism. Most stem cells are in an undifferentiated state and have the potential for long-term division and accumulation, making these cells quite vulnerable to oxidative stress, leading to the generation of cancer stem cells (CSCs) in the population.

**Figure 3:**
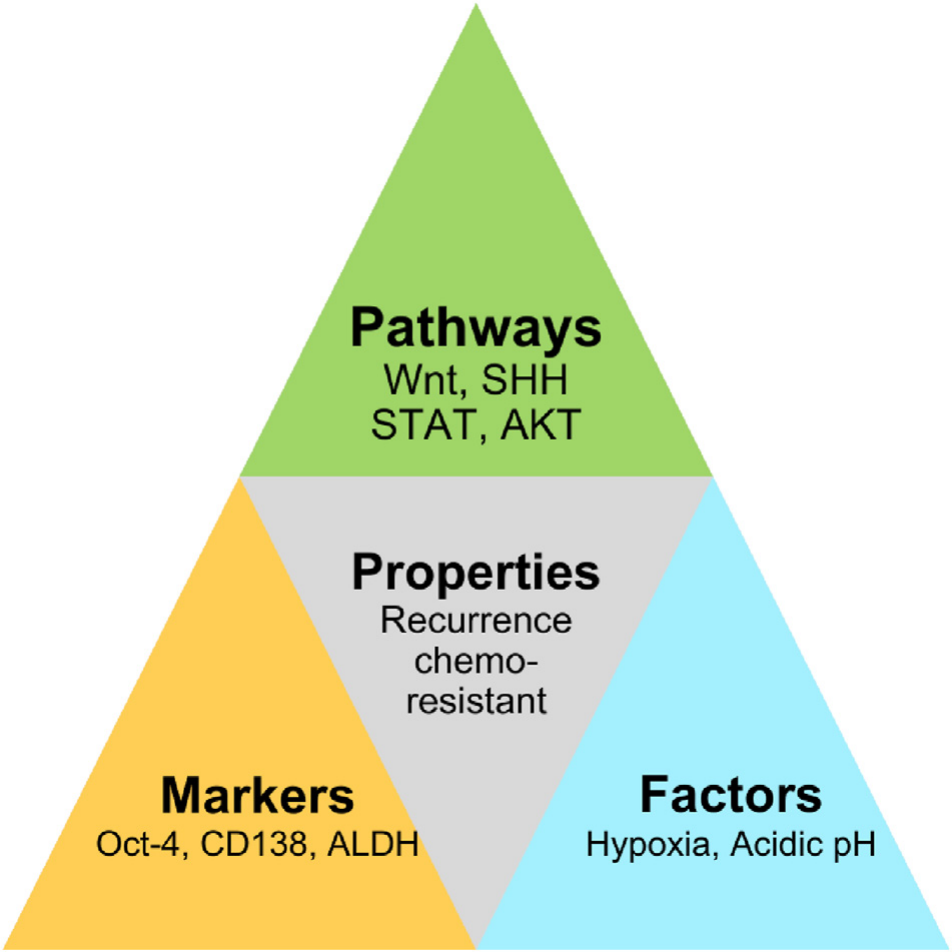
Links between factors, signaling pathways, and surface markers in stem cell-based approaches for OSCC.

In OSCC, CSCs with markers such as *Oct4*, *Sox2*, and *Nanog* are responsible for uncontrolled cell proliferation, self-renewal, and the differentiation capacity, which involve pathways including SHH, WNT, NOTCH, and EGFR (Figure 3).^40^ Targeting CSC-specific markers such as EGFR and CD44 has moderate efficacy in controlling tumor size and the progression of cancer.^41^ Targeting tyrosine kinases in CSCs using EGFR-targeted agents such as cetuximab and erlotinib is moderately effective at arresting tumor progression and the differentiation of OSCC subpopulations. In addition, inhibitors of glutathione synthesis further enhance ROS-based therapeutic effects against CSCs.^16^

## Nanotechnology-based therapy for treatment of OSCC

**Figure 4:**
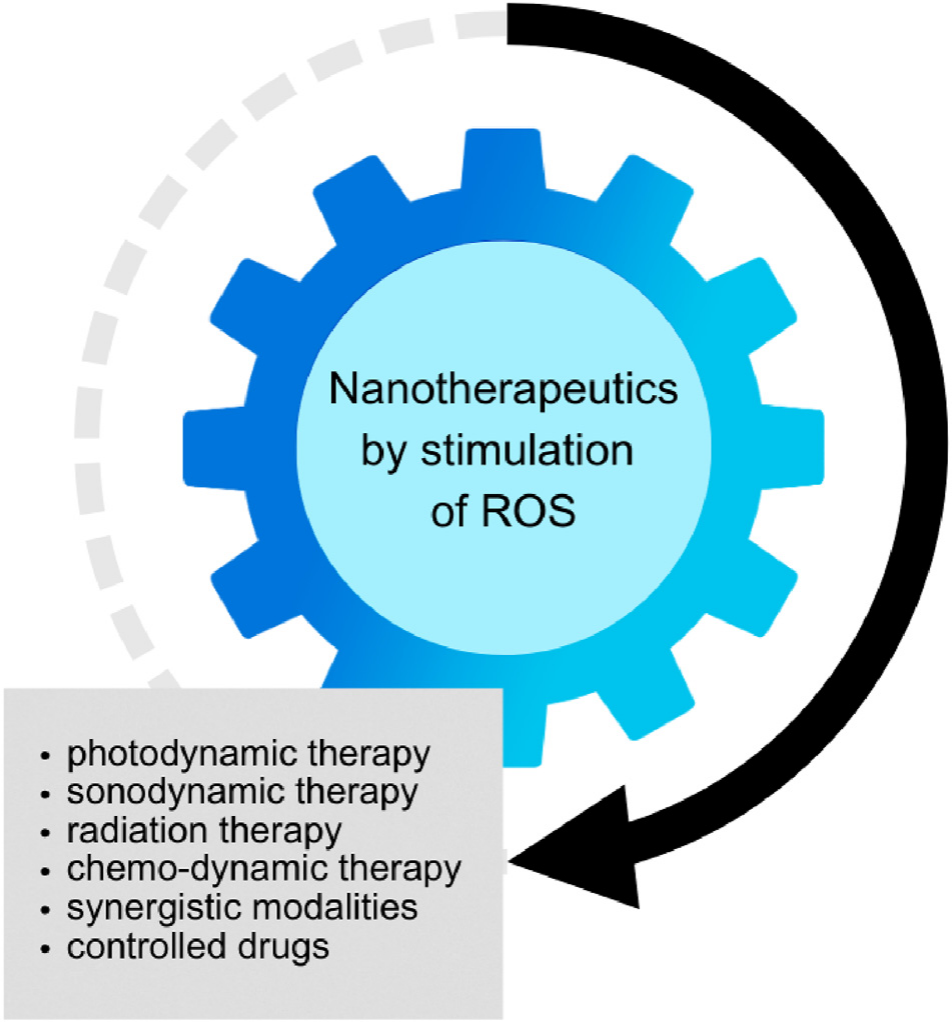
Types of nano-therapeutics used in treatment of OSCC by stimulating ROS.

Nanotechnology approaches such as photodynamic therapy, sonodynamic therapy, and chemo-dynamic therapy combined with ROS upregulation have obtained promising results in enhancing therapeutic outcomes, and they have emerged as viable options for treating OSCC (Figure 4).^42^ However, despite encouraging results, these therapies are affected by challenges including limited oxygen levels and resistance by CSCs.^43^ More innovative medications (small molecule compounds at nanoscale) such as elesclomol exhibit extraordinary therapeutic effects and superior antioxidant capabilities, but their effectiveness and safety for treating different malignancies, particularly OSCC, either in isolation or in conjunction with traditional chemotherapy drugs has not been well explored. Addressing the existing challenges and limitations by using patient-derived xenograft models or improved ROS-based nanomaterials could make these therapies more clinically relevant. Future work should focus on developing cost-effective and accessible strategies to accelerate clinical applications by expanding the testing of widely available nanomaterials.

## Radiation therapy based on ROS-mediated strategies for OSCC

Non-radical (such as hydrogen peroxide and singlet oxygen) and free radical (such as superoxide anion and hydroxyl radical) types of ROS are extremely reactive compounds generated by cellular oxidative metabolism.^44^ Excessive ROS levels can damage proteins, lipids, and nucleic acids, resulting in oxidative stress, even though they are necessary for cellular signaling and homeostasis. ROS levels are usually higher in cancer and they can induce changes in cellular metabolism and the activities of the mitochondrial electron transport chain and NADPH oxidases to promote rapid cell damage, thereby compromising growth and survival.^45^

**Figure 5:**
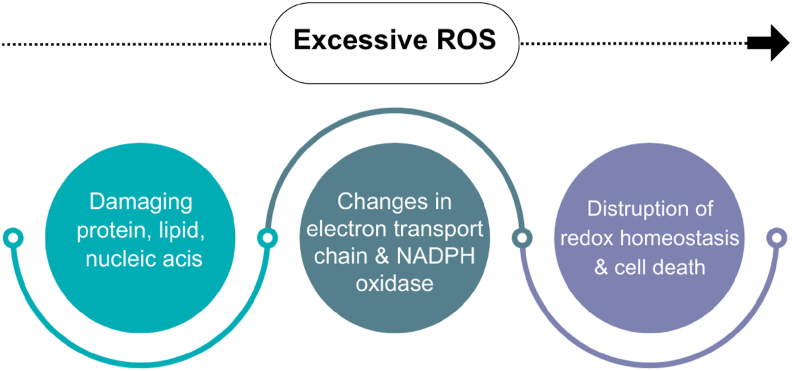
ROS-induced cellular mechanism of radiation-based therapy in treatment of OSCC.

However, the high ROS environment also makes cancer cells vulnerable to ROS-modulating therapies. Cancer cells enhance antioxidant defense systems (e.g., superoxide dismutase and glutathione (GSH)) to manage increased ROS levels, whereas CSCs are resistant. Therapeutic strategies aim to raise ROS levels beyond a tolerable threshold to induce selective cell death in cancer cells (Figure 5). Targeting redox pathways (e.g., Nrf2/Keap1 and Trx) by using emerging ROS-enhancing therapies (e.g., photodynamic therapy) can improve the efficacy of chemotherapy or radiotherapy, with precision monitoring of ROS levels to obtain optimal outcomes.^46^

## Combinational therapy for OSCC

Radiation therapy is a useful treatment option for oral cancer patients because it produces ROS and oxidative stress, which ultimately kill tumor cells. In addition to causing ferroptosis, autophagy, and apoptosis, oxidative stress modifies the immunological response against tumors. The treatment of several malignancies has been completely transformed by the combination of radiation therapy with immunotherapy. The oxidative stress caused by ROS is essential for this process. Radiation therapy-induced ROS can alter the tumor immunological microenvironment, control immune cell infiltration and differentiation, boost the release of tumor-associated antigens, and modify the expression of immune checkpoints.^25^

## Pro-oxidant agents in clinical studies of OSCC

At present, various drugs including phytochemicals and small molecules are being investigated in both pre-clinical and clinical studies due to their anticancer activities, mainly ROS induction. In addition to pre-clinical studies, the selected articles highlighted a few pro-oxidant drugs and compounds that have proceeded into clinical trials (Table 3). The chemotherapeutic drugs that have been clinically tested for treating OSCC include cisplatin, doxorubicin, and curcumin, and they induce ROS as one of the mechanisms of action.^47^ Understanding drug-/chemically-induced oxidative stress is crucial for improving anti-cancer benefits and broadening therapeutic applications because pro-oxidant therapies have resulted in significant adverse effects in pre-clinical research and clinical settings due to the non-specific effects of oxidative stress damage on organs and functional disabilities. Thus, more research is required to manage the side effects and therapeutic resistance caused by oxidative stress to effectively exploit the medicinal potential of pro-oxidants. Consequently, it is important for the scientific community to balance cytotoxic effects and the deleterious effects of oxidative agents to optimize the benefits of pro-oxidant therapy, as well as reducing adverse effects and increasing life expectancy.

**Table 3:** Studies performed using pro-oxidative agents in clinical trials.

Type of pro-oxidative agent	Strategies used	Clinical trial	Reference
		Phase ID	
Cisplastin	Prevention of DNA binding and cross-linking leading to inhibition of DNA replication and transcription	Phase I/II, NCT03502148	48
Doxorubicin	Stops upregulation of miR-221	Phase III **NCT00003888**	49
Curcumin	Superoxide dismutase 1, catalase, glyoxalase 1, and NADPH dehydrogenase [quinone] 1 inhibition.	Phase I NCT01160302	50
		Phase II NCT04208334	51

## Quality assessments of studies

In the present review, simple quality assessments were performed using RoB tools by focusing on the quality of the reported studies, as depicted in Table 4, Table 5, Table 6, Table 7, Table 8. According to the second type of quality assessment (Table 4), among the 15 items, six (40 %) were adequately reported with low RoB, and there was strong evidence of appropriate methodological approaches and minimal risks of inconsistency, reliability and reproducibility. The RoB was moderate for the remaining nine items, but other relevant issues included low reproducibility, small sample sizes, unclear sampling methods, and limited clinical validation.

**Table 4:** Second type of quality assessment.

Study ID	Study Type	Overall Risk of Bias	Key Issues	Comments
NCT03502148 (cisplatin)	RCT	Moderate	Measurement bias	Well-conducted trial but some issues with measurement methods
NCT00003888 (doxorubicin)	RCT	Moderate	Missing outcome data, selective reporting	Some missing data affected reliability
NCT01160302 (curcumin)	RCT	Low	None	Strong methodology and minimal risk
NCT04208334 (curcumin)	RCT	Moderate	Deviation from intended interventions, measurement bias	Some deviation from protocol reported
Goldberg et al., 2022 (cisplatin)	Non-RCT	Moderate	Confounding bias, outcome measurement bias	Confounding bias due to patient selection
Du et al., 2017 (doxorubicin)	Non-RCT	Moderate	Selection bias, missing data	Unclear selection process affected reliability
Moore-Medlin et al., 2015 (curcumin)	Non-RCT	Low	Minor issues	Reliable study with minor concerns
Kim et al., 2012 (curcumin)	Preclinical (animal)	Moderate	Detection bias, reporting bias	Limited sample size
Balakrishnan et al., 2012 (Ferulic acid)	Preclinical (animal)	Moderate	Selection bias, performance bias	Possible inconsistencies in intervention
Xue et al., 2020 (plumbagin)	Preclinical (animal)	Low	None	Strong study with well-controlled variables
Siddiqui et al., 2017 (piperine)	Preclinical (animal)	Low	Performance bias, reporting bias	Reporting bias and performance bias noted
Woo et al., 2016 (metformin)	*In-vitro*	Moderate	Data reliability, external validity	Reliable but limited translation to clinical use
Zhang et al., 2012 (arsenic trioxide)	*In vitro*	Moderate	Reproducibility	Some concerns about reproducibility
Fernandez-Gil et al., 2019 (melatonin)	*In vitro*	Low	None	Well-conducted with strong external validity
Hoshikawa et al., 2011 (d-allose)	*In vitro*	Low	Study design quality	High ROS production noted but limited clinical validation

**Table 5:** Quality assessment for preclinical studies (animal) – SYRCLE’s RoB.

Study ID	Selection Bias	Performance Bias	Detection Bias	Attribution Bias	Reporting Bias	Overall Risk of Bias
Kim et al., 2012 (curcumin)	Low	Low	Moderate	Low	Moderate	Moderate
Balakrishnan et al., 2012 (Ferulic acid)	Moderate	Moderate	Low	Low	Low	Moderate
Xue et al., 2020 (plumbagin)	Low	Low	Low	Low	Low	Low
Siddiqui et al., 2017 (piperine)	High	Moderate	Moderate	Low	Low	Low

**Table 6:** Quality assessment for clinical RCT studies – Cochrane RoB 2.0.

Study ID	Randomization Bias	Deviation from Intended Interventions	Missing Outcome Data	Measurement Bias	Selective Reporting	Overall Risk of Bias
NCT03502148 (cisplatin)	Low	Low	Low	Moderate	Low	Moderate
NCT00003888 (doxorubicin)	Moderate	Low	Moderate	Low	Moderate	Moderate
NCT01160302 (curcumin)	Low	Low	Low	Low	Low	Low
NCT04208334 (curcumin)	Low	Moderate	Low	Moderate	Low	Moderate

**Table 7:** Quality assessment for clinical non-RCT studies – ROBINS-I.

Study ID	Confound-ing Bias	Selection Bias	Deviation from Intended Intervention	Missing Data	Outcome Measure-ment Bias	Reporting Bias	Overall Risk of Bias
Goldberg et al., 2022 (cisplatin)	Low	Moderate	Low	Low	Moderate	High	Moderate
Du et al., 2017 (doxorubicin)	High	Low	Low	Moderate	Low	Moderate	Moderate
Moore-Medlin et al., 2015 (curcumin)	Moderate	Low	Low	Low	Low	Low	Low

**Table 8:** Quality Assessment for *in vitro* studies – ToxRTool.

Study ID	Study Design Quality	Reproducibility	Data Reliability	External Validity	Overall Risk of Bias
Woo et al., 2016 (metformin)	High	High	Moderate	Low	Moderate
Zhang et al., 2012 (arsenic trioxide)	Moderate	Moderate	High	Low	Moderate
Fernandez-Gil et al., 2019 (melatonin)	Low	Moderate	Low	Low	Low
Hoshikawa et al., 2011 (d-allose)	Moderate	Low	Low	Low	Low

Further analyses were performed using Cochrane RoB 2.0 for clinical RCT Studies, ROBINS-I for clinical non-RCT studies, SYRCLE’s RoB for preclinical studies (animal), and ToxRTool for *in vitro* studies. The RoB tool for animal studies was applied to four studies (Table 5). These studies were characterized by five types of bias: selection bias, performance bias, detection bias, attribution bias, and reporting bias, as well as overall RoB. Item 1 matches with items 3 and 4 in the Cochrane RoB tool (Table 6). In addition, the ROBINS-I assessments for non-RCTs highlighted one entry with a high risk of reporting bias in a study of cisplatin, and the other two studies were characterized by low to moderate risk (Table 7). The ToxRTool was used to assess the RoB for *in vitro* studies considering aspects such as study design, experimental procedures, and data analysis (Table 8). The ToxRTool results indicated low to moderate risk of overall bias. However, one study had a high RoB according to the study design quality and reproducibility due to issues with data reliability, external validity and limited translation to clinical use. Significant differences were found in the *in vitro* studies, animal studies, and clinical studies in terms of the types of pro-oxidants used. Most of the differences detected by RoB tools were due to differences in research design between RCTs, non-RCTs, *in vitro* studies, and animal studies. Shortcomings or unfamiliarity with specific aspects of the experimental design of laboratory studies compared with clinical studies may also have contributed to these differences.

## Comparisons with previous scoping reviews of pro-oxidant therapies for OSCC

In addition to analyzing the 15 selected studies, we compared our findings with previous systematic or scoping reviews performed on similar topics. The potential of pro-oxidant therapy for OSCC treatment is supported by a few recent studies, which highlighted its efficacy and mechanisms. Hence, we explored the similarities and differences among previously published recent reviews and the present review of pro-oxidant therapy in OSCC, as well as the mechanisms and challenges associated with its application. Similar to the pro-oxidant agents highlighted in our findings, ROS induction can be achieved at the cellular level by using erufosine to either directly generate ROS or disrupt the redox balance within cancer cells.^52^, ^53^, ^54^ Targeting mitochondrial pathways can enhance the efficacy of pro-oxidant therapy by inducing mitochondrial dysfunction and promoting cancer cell apoptosis.^54^ Pro-oxidant therapy can effectively target cancer cells but there is a risk of collateral damage to normal tissues due to uncontrolled oxidative stress, thereby necessitating careful monitoring and balancing of ROS levels.^52^ Another interesting review article considered the potential of using small molecule redox active agents, specifically melatonin, vitamin E, selenium, and vitamin C, to act as pro-oxidants in cancer cells while maintaining the antioxidant properties of normal cells.^55^ However, the use of antioxidants during cancer treatment can potentially interfere with the efficacy of pro-oxidant therapies, which emphasizes the importance of maintaining the redox balance in cancer treatment.^56^ Therefore, identifying reliable biomarkers of oxidative stress, such as malondialdehyde, and assessing the responses to treatment are crucial for optimizing pro-oxidant therapy and personalizing treatment plans.^57^

## Limitations of current review and future directions

The number of studies included in our scoping review was low but this exploratory review may help to provide a clear map of existing studies and evidence to support concise systematic reviews in the future, and thus facilitate the development of new policy and practices. However, it is important to note that directly comparing the studies presented in this review is challenging because they employed different experimental models and methods. Most pro-oxidant medicines are still in the experimental stage and there is a lack of extensive phase III trials to validate their long-term safety and efficacy, even though we selected publications from both clinical and preclinical trials. Furthermore, the relevance of our findings is limited by the diversity of OSCC patient populations, which vary in terms of their tumor microenvironments and genetic backgrounds.

Pro-oxidant therapy is a potential treatment option for cancer because cancer cells have a higher base level of ROS than normal cells, which makes them more susceptible to oxidative damage. Pro-oxidant therapies have obtained promising results, but they are also limited by factors such as tissue penetration, limited bioavailability, and resistance mechanisms, and thus more clinical data are needed. Clearly, unregulated or indiscriminate oxidative stress might harm most cells, including normal cells, which will negatively impact the prognosis and survival rate of cancer patients. Pro-oxidant therapy may also limit the population of cancer cells with stem cell characteristics to prevent metastasis and cancer recurrence. However, the detrimental effects of excessive ROS generation, which might harm healthy cells, have not been investigated widely in clinical studies as they may compromise patient safety and raise ethical concerns.

Despite the growing interest in introducing more pro-oxidant agents for treating OSCC, the lack of comprehensive data from animal studies and clinical data hinders assessments of the potential efficacy of these compounds. Therefore, we recommend that the current approaches are refined in future research, with the primary aim of enhancing targeted delivery to generate ROS in targeted cancer cells and improve patient responses. Advances in drug formulation and nanotechnology can also help enhance penetration into deeper tissues and bioavailability to increase the likelihood of these therapies being practically useful in clinical setting.

Previous studies also highlight the need to focus on standardizing experimental protocols to improve the reproducibility, reliability, repeatability, and quality of pro-oxidant therapies. Ideally, the efficacy of pro-oxidant therapies for OSCC patients can be increased by implementing well designed laboratory and clinical trials. Combining pro-oxidant agents with immunotherapy, chemotherapy, or radiation therapy can increase their synergistic effects. Pro-oxidant therapy represents a promising avenue for OSCC treatment but it is essential to consider the potential for adverse effects and the complex interplay between pro-oxidant and antioxidant mechanisms.

Further research is needed to refine pro-oxidant therapies, develop reliable biomarkers, and explore combined strategies that can enhance the efficacy while minimizing harm. The integration of pro-oxidant therapy with existing treatment modalities could potentially improve outcomes for OSCC patients but careful consideration of the underlying biological mechanisms and patient-specific factors is required. Thus, additional research is also needed to maximize the dosage and reduce damage to healthy cells by focusing on toxicity and side effects. Finally, other type of pro-oxidant treatments, such as metabolic inhibitors, dietary antioxidants, and gene-editing techniques, could potentially be explored to increase the range of therapeutic possibilities for treating OSCC.

## Conclusion

The present scoping review has several strengths, particularly mapping the scope and nature of research into the development of pro-oxidant therapy for OSCC. The advantages of this review include comprehensive literature search and database screening, identifying existing knowledge gaps, and developing an important database to support future research and application in clinical practices. The information presented in this review is a valuable tool for exploratory research, especially in emerging areas where previous research may be highly complex or heterogeneous in nature. The comprehensive literature search was performed by following specific rules based on clear inclusion and exclusion criteria, and the strict selection of high-quality articles improved the reliability of our findings.

The present scoping review also describes categories of pro-oxidant therapies that have been tested in previous research based on a variety of approaches, including nanotechnology-based, stem cell-based, phytocompound-based, radiation-based, and combination therapies, thereby providing a well-structured understanding of the therapeutic landscape of OSCC. Integrating findings from *in vitro* and *in vivo* investigations with clinical trial data facilitated the identification of research gaps in different studies. In addition, we discussed evidence-driven strategies that strengthen pro-oxidant treatment approaches, such as by focusing on oxidative stress in CSCs and the application of nanotechnology, which are important for the future development of OSCC therapy.

## Ethical approval

Not applicable.

## Authors contributions

NY designed the initial study and finalized the final manuscript. ZN conducted research, analyzed research material, and organized and interpreted data. NI, HM, and MDM proofread and critically reviewed the final draft. All authors have critically reviewed and approved the final draft and are responsible for the content and similarity index of the manuscript.

## Source of funding

This research was supported by the 10.13039/501100002385Ministry of Higher Education (MOHE) Malaysia through the Fundamental Research Grant Scheme (FRGS/1/2020/SKK06/USM/03/3).

## Conflict of interest

The author(s) have no conflict of interest to declare.
